# Effects of Dietary Fat Profile on Gut Microbiota in Valproate Animal Model of Autism

**DOI:** 10.3389/fmed.2020.00151

**Published:** 2020-05-12

**Authors:** Jin-peng Wang, Yang-chun Xu, Ji-qiu Hou, Jia-yu Li, Jie Xing, Bao-xia Yang, Ze-hui Zhang, Bei-lin Zhang, Hong-hua Li, Ping Li

**Affiliations:** ^1^Department of Cardiology, The Second Hospital of Jilin University, Changchun, China; ^2^Department of Dermatology, The Second Hospital of Jilin University, Changchun, China; ^3^Department of Pharmacy, The Second Hospital of Jilin University, Changchun, China; ^4^Department of Physiology, College of Basic Medical Sciences, Jilin University, Changchun, China; ^5^Department of Developmental Pediatrics, The Second Hospital of Jilin University, Changchun, China; ^6^Department of Developmental and Behavioral Pediatrics, The First Hospital of Jilin University, Changchun, China

**Keywords:** autism spectrum disorder, gastrointestinal, fatty acid, microbiota, behavior, valproate acid

## Abstract

Autism spectrum disorder (ASD) is a developmental disability which may cause significant social, communication, and behavioral challenges. Besides certain essential symptoms, a lot of ASD individuals also suffer the comorbidity of gut microbiota dysbiosis, which possibly causes a variety of gastrointestinal (GI) difficulties. Interestingly, evidence has indicated that behavioral output may be modulated through the communication between the central nervous system and gut microbiota via the gut-brain axis. Polyunsaturated fatty acids (PUFAs) and n-3 fatty acids (n-3 PUFA) are structurally and functionally crucial components for the brain, and the state of n-3 PUFAs also affects the gut microbiota. However, how varying intake ratios of n-3/n6 PUFAs affect the gut microbiota composition in ASDs is not well-understood. Pregnant female Wistar rats with intraperitoneal administration of valproate acid (VPA) at embryonic day (E) 12.5 and their male offspring were grouped and fed three diets: a control chow (VPA group), omega-3 deficient (A group), and n-3/n6 (1:5) diet (B group). The diet of pregnant female Wistar rats with intraperitoneal administration of saline and their male offspring was a control chow (normal group). Microbial composition and species abundance were investigated accordingly by the 16S rRNA gene-based metagenomics analysis on the fecal samples. Results showed that fecal microbial abundance was decreased because of VPA administration in the period of pregnancy, and the changing pattern of gut microbiota was similar to that reported in ASD patients. Furthermore, the n-3/n6 (1:5) diet increased the fecal microbial abundance and decreased the elevated Firmicutes. In conclusion, n-3/n6 PUFAs (1:5) diet supplementation may alter gut microbiota composition in VPA-exposed rats. This study put forward a new strategy for the intervention and treatment of autism by n-3/n-6 PUFAs ratio supplementation intakes.

## Introduction

Autism spectrum disorder (ASD) includes a variety of neurodevelopmental disorders and continuously shows defects in social communication and social interaction in various environments, a limited, repetitive act, interest, or activity (The Diagnostic and Statistical Manual of Mental Disorders Fifth Edition, DSM-V). The interaction between the human gut and brain is complicated, and gut dysbiosis may make individuals susceptible to neurodevelopmental disorders. It is known that the ASD population may suffer long-term gastrointestinal (GI) symptoms as commensal common medical conditions, such as abdominal cramps, constipation, diarrhea, suggesting that there is a relationship between GI microbiota and the development and therapy of autism (1).

The power of gut microbiota can be seen in numerous physiological fields. It is thought that the development of the immune system, gut barrier functioning maintenance, and GI motility regulation are dependent on intestinal bacterial colonization. Gut microbiota is regarded as a leading force in host development and the functions of organisms, and such acknowledgment now reaches out to the core characteristics of the brain and behavioral patterns. From the moment an individual is born, the establishment of the microbial composition begins, and the microbiota are subjected to changes due to the manner of infant birth delivery and dietary habits. The implication of changes (including gut microbiota composition, gut microbiota metabolites, the communications between gut microbiota and human brain) reflects the complicated pathophysiology of ASD (2). A variety of recent studies have assessed the role of gut microbiota in ASD (3). Very little is known about the potential mechanisms, as GI symptoms are commonly commensal in ASDs.

Rodent models applied in human ASD have been put forward as (A) Natural Models: rodent strains with characterized behaviors related to ASD syndromes; (B) ASD-relevant genetic mutation Models; and (C) Acquired behavior Models: the insulting results of varying environments either having direct effect on the development of an animal or having an indirect effect on the mother who has ASD descendants. In one of the prenatal-induced models, the medical condition of the pregnant rats with valproic acid (VPA) administration exposed its offspring's ASD-like behaviors, which had connections with changes in gut microbiota related to various inflammations and endocrines in the intestinal canals as well as the nerve system (4). Some studies have reported that children with both ASD and GI problems present more serious autism or autism-relevant symptoms than ADS children without GI problems, and there is doubt on whether certain GI problems may result from gut microbiota dysbiosis (5). Given the fact that GI disorders are treatable, it is ascertainable to improve ASD-relevant symptoms or behavioral patterns by treating GI disorders (6).

A lot of children with ASD also have GI pathological changes, such as increased permeability of intestines, an infectious gut with Clostridium difficile produced by cresol, and general alterations of microbiota (7). Intestinal microbiota transplantation (IMT) can be regarded as a promising method to treat GI disorders in children with ASD by transplanting healthy fecal microbiota from donors with efforts to restore a sound and well-functioning microbiota composition (8). Studies have reported the alternations of gut microbiota in ASDs qualitatively and quantitively. The characteristics of such gut microbiota dysbiosis include the increased Clostridium species and genus-level Desulfovibrio, the decreased bifidobacteria, and the translocation of intestinal bacteria (9).

The dysbiosis of intestinal bacteria presented in the VPA-induced rat autism model is similar to that of human autism (10, 11). Oral administration with human symbiotic Bacteroides fragilis in the autism rat model of the maternal immune activation (MIA) can improve the intestinal permeability and change the microbic composition. Impaired behaviors in communication and interaction are also ameliorated, such as restricted, repetitive, dysphoric, and sensomotor patterns of behaviors (1). In the ASD animal model, the serum metabolomic profile is altered, and *B. Fragilis* may regulate the levels of some metabolites. Certain abnormal behaviors of immature rats result from treatments using a metabolite, which is regulated by MIA and recovered by *B. Fragilis*, suggesting that the influence of intestinal bacteria on the host metabolome can affect behaviors (1). MIA-mediated changes in 4-ethylphenyl sulfate (an organism-dependent metabolite inducing dysphoric behaviors) can be corrected by the therapy. In addition, the pathological composition of intestinal symbiotic strains in the ASD naive rats may be corrected by the probiotic's treatment. Probiotics can be colonized in the intestinal tracts and can enter the blood circulation through metabolites regulated by microorganisms, thus affecting the behaviors of ASD rats and improving the relevant symptoms. Therefore, from the perspective of gut microbiota, interventions with the major functional bacillus genus related to autism, may serve as a strategy to prevent and/or treat autism by modulating the gut microbiota composition, which may also elucidate the potential mechanism of ASD progression in children.

In recent years, increasing attention has been paid to the metabolic abnormality of autistic children. Studies have revealed decreased levels of polyunsaturated fatty acids (PUFAs) and abnormalities of metabolic pathways in children with ASD (12, 13). Considering the crucial role of PUFAs in the development of the nervous system, to investigate the relationship between PUFAs and ASD pathogenesis has become a new hot topic in the field of neurology.

PUFAs are straight-chain fatty acids containing more than one unsaturated double bond in their backbone with 16–22 carbon atoms. According to the number of double bond positions starting from the hydrophobicity of the fatty acid carbon chain, PUFAs can be classified into various groups, including n-3, n-6, n-7, and n-9, where n-3/n-6 PUFAs are crucial for the development and functions of the brain and nervous system (14). The central nervous system (CNS) is rich in n-6 and n-3 PUFAs, specifically docosahexaenoicacid (DHA; n-3) (a crucial PUFA in the development of a baby's brain, accounting for 20% of dry weight of the brain approximately), and arachidonic acid (AA; n-6).

n-6 and n-3 PUFAs mutually interact and confer synergetic effects in the human body. The dietary ratio of n-6/n-3 PUFAs intake is about 1-2:1 as humans evolve. Nevertheless, in modern western countries, the ratio of n-6/n-3 PUFAs intake in the diet is up to 20–30:1 (15). In various observational studies, many mental disorders like ASD are relevant to the imbalance of n-3/n-6 PUFAs, suggesting that the imbalanced n-6/n-3 intake ratio in diets may cause mental sensorimotor disorders in youth, and even impose intergenerational influence (16).

During the early postpartum period, brain development is closely related to the complex gut microbiota in the human body. Increasing evidence has shown an indispensable connection between brain and gut microbiota. Given the powerful interactions between the gut microbiota and diets, regulating the composition and functions of gut microbiota by dietary adjustment may be employed to regulate the behaviors and brain biochemistry (17). Moreover, n-3 PUFAs supplementation may improve disturbances in the gut microbiota caused by the pressures in early life (18).

The present study aimed to investigate the influence of varying n-6/n-3 PUFAs dietary ratios on the gut microbiota in an early life rat model of ASD. The mother rats were parentally treated with 400 mg/kg VPA at embryonic day (E) 12.5, and their offspring were used for further investigations.

## Materials and Methods

### Animals

A total of 25 9-week-old female Wistar rats were purchased from Changchun Yi Si Laboratory Animal Technology Co., Ltd. (SCXK-[JI] 2016-003, Jilin, China). Animals were given *ad libitum* access to water and food, and the room temperature was maintained at 20 ± 1°C with a 12 h:12 h light/dark cycle. Mating commenced 7 days after rats were moved to their new dwellings. All the procedures were performed according to the National Animal Care Guidelines, and this study was approved by the Ethics Committee of Jilin University (2019071).

### VPA Administration

The pregnant rats were intraperitoneally treated with VPA (400 mg/kg, Sigma P4543) at E12.5 (*n* = 10). Sterile saline was injected intraperitoneally for pregnant rats in the control group at E12.5 (*n* = 4). From day 22 to 12 weeks after birth, male offspring were grouped and fed a n-3 deficient diet, standard control chow, or a n-3/n-6 PUFA = 1:5 diet.

### Diets

There were three types of diets used in the present study: (1) standard control chow: 4.0% fat, 8.0% ash, 21% protein, 5.0% fiber, and 47% complex carbohydrates; (2) n-3 deficient diet (A group); (3) n-3/n-6 PUFA = 1:5 diet (B group). The three dietary provisions were provided by Beijing HUAFUKANG bioscience Co, Inc in China, and the macronutrient contents of diets were all the same, only the fatty acid profile was different. A detailed macronutrient list, and the corresponding fatty acid compositions are shown in Table 1. Considering the risk for the oxidation of long-chain n-3 PUFAs, airtight storage packing was used, and all diets were stored in the dark at 4°C.

**Table 1:** Diet macronutrient profiles.

**Ingredients of the diet (g/kg)**	**Control**	**A (n3 free)**	**B (n3/n6 = 1:5)**
Casein	200	200	200
Cornstarch	388	388	388
Sucrose	150	150	150
methionine	3	3	3
Sunflower seed oil	0	660	110
Vitamin mixture	10	10	10
Mineral mixture	35	35	35
Cellulose	47	47	47
Choline	2.5	2.5	2.5
Calcium	4	4	4
lard	0	300	300
Linseed oil	0	0	40
Fish oil	0	0	510

### Experimental Design

The male offspring of rats were subsequently divided into four groups: (1) the normal group (*n* = 5) with control standard chow (whose mothers were administrated saline at E12.5); (2) the VPA group (*n* = 6) with control standard chow (whose mothers were administrated VPA at E12.5); (3) the A group (*n* = 5) with type A chow (whose mothers were administrated VPA at E12.5); and (4) the B group (*n* = 4) with type B chow (whose mothers were administrated VPA at E12.5).

### DNA Extraction

At 12 weeks after birth, male offspring were transferred individually to fresh sterilized cages, and their feces were sampled from the rectum into pre-labeled microfuge tubes, immediately flash frozen in liquid nitrogen and then stored at −80°C. A QIAamp 96 PowerFecal QIAcube HT kit (QIAGEN, Germantown, MD, USA) was used to extract the DNA of microbes in the sampled feces. A BioAnalyzer 2100 (Agilent, Palo Alto, CA, USA) was used to quantify the integrity and concentration of DNA. Before PCR amplification, extracted DNA was stored at −80°C.

### Illumina Sequencing of 16S rRNA Genes

In accordance with the instructions of the 16S metagenomic sequencing library protocol (Illumina), the generation of V3-V4 amplicons was done for the purpose of Illumina sequencing. In brief, specific primers were utilized for initial PCR amplification of the V3-V4 region of the 16S rRNA gene, with 343F, 5′- TACGGRAGGCAGCAG−3′ and 343F, 5′- TACGGRAGGCAGCAG−3′ as forward primer and reverse primer, respectively.

The conditions for PCR amplification were as follows:

94°C for 5 min;
94°C for 30 s with 26 cycles;
56°C for 30 s;
72°C for 30 s;
72°C for 5 min;
storage at 4°C.

PCR products were subjected to electrophoresis in 1% agarose gels, and a Takara Ex Taq [Takara Biomedical Technology (Beijing) Co., Ltd.] was utilized for the purification of PCR products. Then, the Illumina MiSeq (Illumina, San Diego, CA, USA) was used to sequence the V3-V4 PCR products.

### Bioinformatics

Trimmomatic software was employed for the preprocessing of paired-end reads to examine and block off ambiguous bases (N). A sliding window trimming approach was also implemented to cut off the sequences with poor quality (scored quality average under 20). FLASH software was then utilized to assemble paired-end reads after the completion of sorting (19).

The setting of assembling parameters was:

Overlapping: 10 bp (minimum), 200 bp (maximum)
Mis-pairing rate: 20% (maximum)

Further sequencing was implemented for denoising. Reads with 75% of bases over Q20 were preserved, while the reads whose sequence was unclear, similar, or under 200 bp were excluded. Further, detection and removal of reads with chimera was also performed. 1.8.0 versioned QIIME software was applied to realize the above procedures (20). Using Vsearch software, primer sequence removal and assembling of “Cleared” reads were performed for the generation of operational taxonomic units (OTUs) with a similarity cutoff rate at 97% (21). The selection of characterized reads of all OTUs was conducted with the QIIME kit. The RDP classifier with a 70% threshold confidence was utilized to mark and blast each characterized read against Greengens, or the 123 versioned Silva database, or 16s rDNA (22).

### Statistical Analysis

Microbial data was normalized and uploaded into MetaboAnalyst 3.0 for modeling using partial least squares discriminant analysis (PLS-DA). The quantitative data are expressed as mean ± standard deviation (SD). One-way variance analysis was performed to measure the statistical deviations in each group. A value of *P* < 0.05 was considered statistically significant. The algorithm of Linear Discriminant Analysis Effect Size (LEfSe) was applied so as to recognize relative abundant values of OTUs as well as pathways exhibiting significant deviations under two kinds of biological environments which would be subjected to a defaulted cutoff in accordance with ranking of Kruskal-Wallis test (*P* < 0.05 and LDA > 2.0 or the score of absolute log 10 LDA).

## Results

### Alpha Diversity Analysis

**Figure 1:**
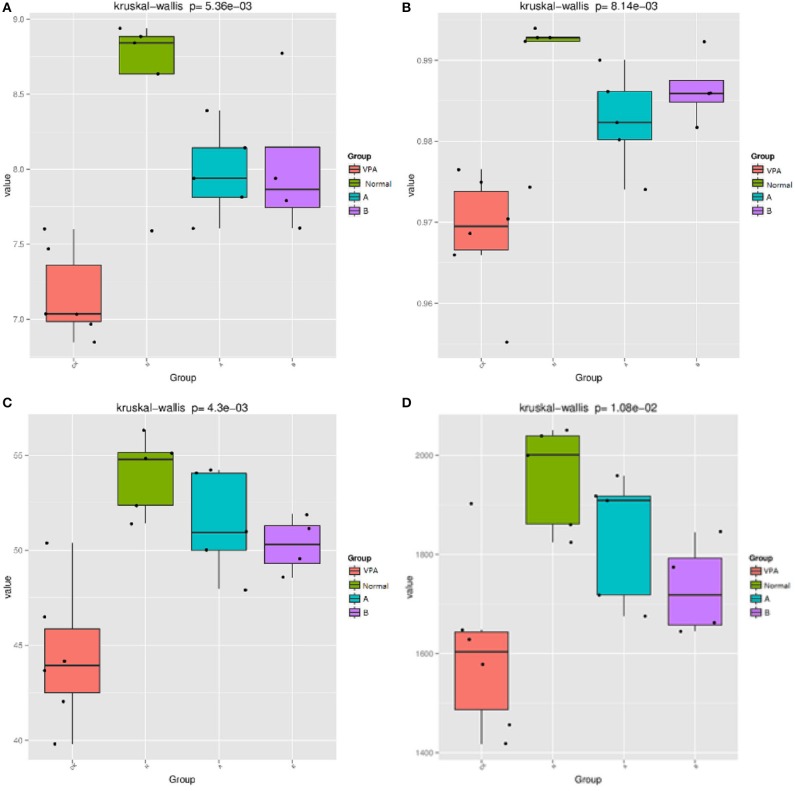
Effects of different diets on THE gut microbiota in αdiversity indices. **(A)** Shannon value; **(B)** Simpson value, **(C)** PD whole tree value; **(D)** Chao1 value. mean standard deviation (SD), *P* < 0.001.

The indices, including Shannon, Simpson, Chao1, and PD whole tree were used to evaluate the phylogenetic diversity. The OTU richness was determined by calculating the Observed Species, Shannon, Simpson, Chao1, and PD_whole_tree indices based on the total number of species. PD whole tree evaluates the phylogenetic diversity while other metrics are used to reflect species diversity. The measured Chao1 value was 1955.23 ± 46.96 (*N* = 5) in the control rats. VPA significantly decreased the Chao1 value to 1605.03 ± 70.389 (*N* = 6, *P* < 0.01, Figure 1D). The Shannon value was 8.58 ± 0.25 in the control rats and 7.16 ± 0.12 in the VPA group (*P* < 0.01, Figure 1A). The PD tree value was 54 ± 0.91 in the control rats, which was lower than that in the VPA group (44.43 ± 1.49, *P* < 0.01, Figure 1C). The Simpson value was 0.99 ± 0.003 in the control group, which was lower than that in the VPA group (0.97 ± 0.003, Figure 1B). These trends were consistent with the change in the abundance of OTUs in the VPA group.

After administration of the dietary supplement, the microbial diversity was significantly improved in both the B group (50.27 ± 0.75, *P* = 0.004) and A group (51.43 ± 1.2, *P* = 0.001) as compared to the VPA group (44.43 ± 1.49), as shown by the PD tree index. The Simpson value in the B group (0.99 ± 0.002) and A group (0.98 ± 0.003) was significantly higher than that in the VPA group (0.97 ± 0.003, *P* < 0.01). The Shannon value in the A group (7.979 ± 0.135) and B group (8.027 ± 0.25) was markedly higher than that in the VPA group (7.16 ± 0.12, *P* < 0.01). Others showed no differences.

### Beta Diversity Analysis

**Figure 2:**
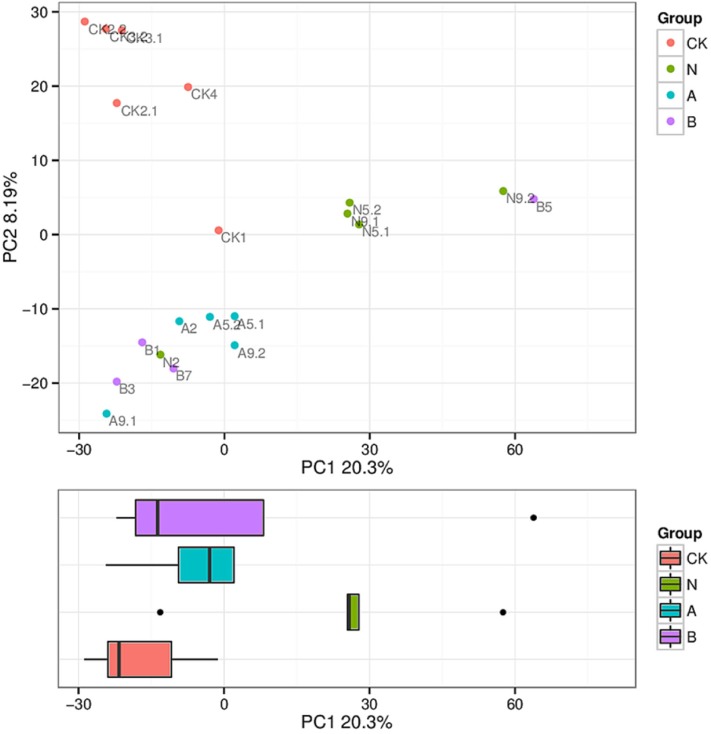
Principle coordinates analysis (PCoA) of microbiota composition. Clustering of microbial communities with PCoA based on 20 fecal samples. The ratio of deviations in accordance with the explanation of principal component is marked on the axes.

The similarity level of various microbial communities was assessed by a Beta diversity analysis. ADONIS with Bray–Curtis similarity index was used to examine the overall microbiome significance. Among four groups, significant deviations (*P* = 0.001, *R* = 0.531) were presented according to the beta diversity analysis, and the difference ratio was 20.3 and 8.19%, respectively, as shown in the explanation of the principal component (Figure 2).

### Differential Analysis of Gut microbiota

**Figure 3:**
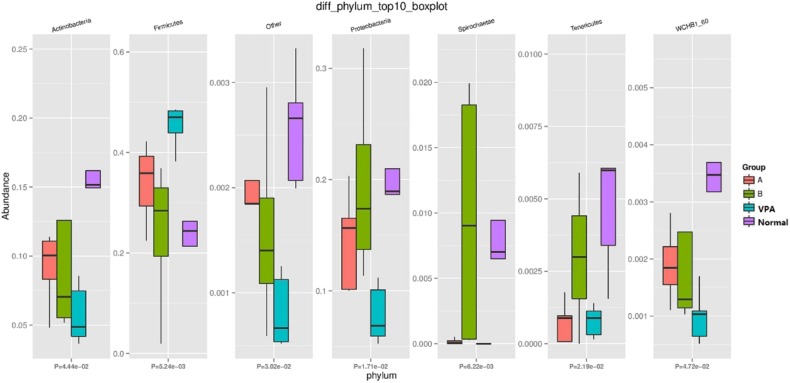
Differential Gut Microbiota at Phylum-level in fecal samples.

At phylum level, the amount of Tenericutes, Firmicutes, Proteobacteria, Fusobacteria, and Spirochaetae was significantly different among the four groups (Figure 3). The number of Firmicutes in the VPA group was increased as compared to the other three groups (*P* = 0.005). Interestingly, species associated with Firmicutes in the B group was similar to those in the control group.

**Figure 4:**
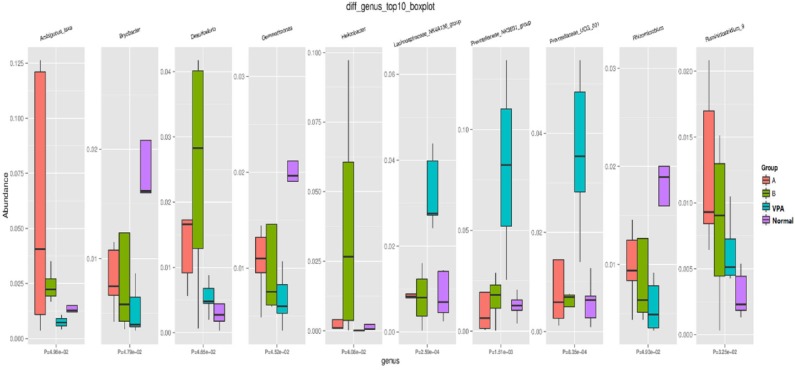
Microbial distributions at Genus-level in the fecal samples of the four groups. Red: high; Blue: low CK as VPA group, N as normal group, A as A group and B as B group.

At genus level, significant differences were found in the Ambiguous, Bryobacter, Desulfovibrio, Gemmatimonas, Helicobacter, Lachnospiraceae, Prevotellaceae, Rhizomicrobium, and Ruminiclostridium among the four groups (Figure 4).

### LDA Effect Size Analysis (LEfSe)

**Figure 5:**
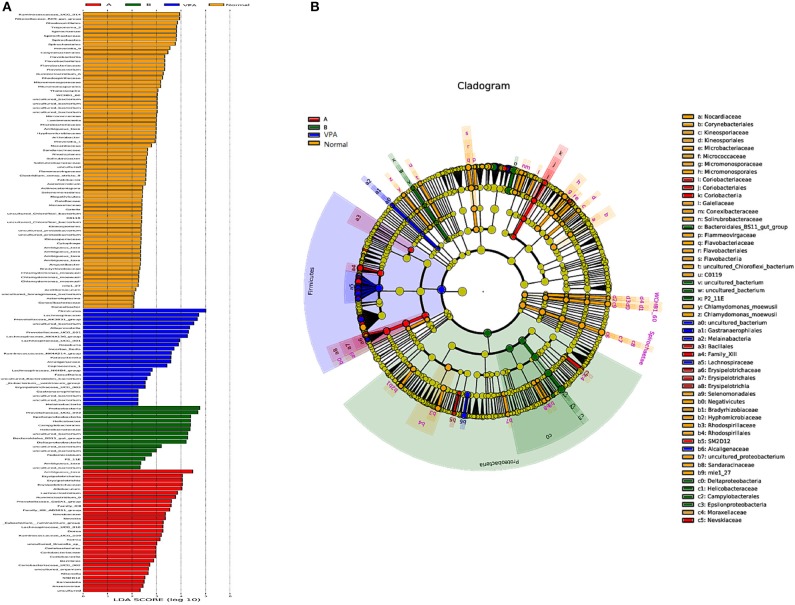
Differential taxa abundance in the gut microbiota after different dietary interventions in the VPA rats. The scores of significantly-altered taxa against different experimental diets through linear discriminate analysis (LDA) are presented in **(A)**. The most abundant taxa following dietary intervention are presented in **(B)** in the manner of cladogram.

LDA effect size analysis (LEfSe) revealed that n-3/n-6 PUFAs (1:5) treated rats had greater taxa abundance in the proteobacteria families, while n-3 PUFAs deficient rats had elevated abundance in the Firmicutes, Coriobacterila (Figure 5).

## Discussion

In the present study, the effects of dietary n3/n-6 PUFAs on the gut microbiota were investigated in an autistic rat model using 16S rRNA sequencing. Our results showed the gut dysbiosis in the VPA group as compared to the control group, while n3/n6 PUFA (1:5) supplementation could ameliorate gut dysbiosis.

In the past decade, the gut microbiota of humans has been extensively and widely discussed. The gut-brain axis has been proposed as a bidirectional neural process of information communication among nerves, endocrine, and the immune system.

Mammals often contain similar phylum-level microbiome abundance. However, the abundance and diversity of microbial species vary significantly among different individuals (23). Many factors have been found to be associated with this difference, such as the environment, diets, diseases, pressures and strains, physical and medical conditions, genetics, and others (24).

Under physiological conditions, the dynamic balance of gut microbiota is important for human health. When the gut microbiota becomes imbalanced, the intestinal functional mucosal barriers may be impaired. The leakage of bacterial amyloid and lipopolysaccharides may increase the permeability of the blood-brain barrier, thus affecting the CNS and causing clinical symptoms and even diseases. The change of intestinal flora in autistic patients mainly, are manifested by the significant increase of intestinal bacterial translocation, the elevated amount of clostridium species, increased Desulfovibrio, and decreased bifidobacteria. Compared with neurotypical children, the intestinal flora abundance is decreased in children with autism (25).

Simpson, PD tree, Chao1, and Shannon's indices were used to evaluate the richness and diversity of the overall microbial community. The microbial community abundance indices mainly include Chao1 and PD tree indices. Microbial community diversity indices include Shannon and Simpson. Our results showed that the microbial community abundance and diversity indices were decreased in the VPA group as compared to the control group, while n-3/n-6 PUFA (1:5) supplementation increased the values of the PD tree, Shannon, and Simpson in the VPA groups. These results suggest that dietary supplementation of n-3/n-6 PUFA (1:5) can significantly improve the abundance and diversity of gut microflora in autistic rats.

Typically, the dominant bacteria in the gut microbiota are Bacteroidetes and Firmicutes (10). Both were also dominant at the phylum-level in the VPA-exposed rats. Some studies on the gut microbiota in mammals have identified bacteria which mostly belong to phylum Bacteroidetes and Firmicutes and the presence of these bacteria are highly susceptible to the dietary influence of the host (26).

Nevertheless, the gut microbiota contains hundreds of discernible bacteria species and almost 90% belong to the phylum Bacteroidetes or Firmicutes as shown by16S rRNA sequencing (27). Increased Firmicutes is characteristic of the gut microbiota in BTBR animals (an autism mice model) (28). Our findings showed significant variance in the amount of Firmicutes, Fusobacteria, Proteobacteria, Spirochaetae, and Tenericutes among the four groups at the phylum-level. The amount of Firmicutes in the VPA group was increased when compared with the other three groups. Following n-3/n-6 PUFA (1:5) supplementation, the amount of Firmicutes was similar to that in the control group. As one class of Firmicutes, the differential subdivision of Clostridia can be up to 20 clusters approximately, and both *C. leptum* and *C. coccoides* belong to the genus Clostridium which is abundant and can generate short-chain fatty acids (SCFAs) (29, 30). As shown in recent studies, the communication between the brain and SCFAs butyrate and propionate is active (31). Our results indicated that n-3/n-6 PUFAs (1:5) supplementation improved the behavioral patterns of autistic rats by reducing Firmicutes in their intestines.

Sun et al. found that the average abundance of Prevotellaceae in the ASD group was lower than in healthy children (25). Our findings showed that the abundance of Prevotellaceae in the VPA group was lower than that in the control group at the genus level. Furthermore, abundance of Prevotellaceae in animals who received a n3/n6 (1:5) supplemented diet was higher than that in the VPA group at the genus level.

Concern about the influence nutrients have on brain health and behaviors is growing. The course of the gut microbiota development overlaps that of intestinal barriers (32). Gut microbiota is one of the factors affecting the integrity of the gut barrier, because gut microbiota can regulate the functions of intestinal barriers by altering the distribution and expression of tight junction proteins (33). Given the functional role of gut microbiota in the behaviors of ASDs, the investigations focus on the microbiota-gut-brain axis (34).

As shown in some recent studies, the change in gut microbiota composition may affect gut permeability, leading to the entry of LPS in the circulation and therefore increasing the risk of long term low-grade systemic inflammation (35). There is evidence showing that n-3 and n-6 PUFAs can improve the behaviors of autism by improving gut microbiota therefore reducing the inflammatory exudation of the intestines. Our study also confirmed that dietary supplementation of n3/n6 (1:5) significantly changed the composition of gut microbiota in the VPA treated rats. We also detected the behaviors of rats and the results were published elsewhere. Therefore, supplementation of n-3/n-6 PUFAs at an appropriate ratio in diets may serve as a new strategy for the intervention and treatment of autism.

## Data Availability Statement

The datasets analyzed in this article are not publicly available. Requests to access the datasets should be directed to Ping Li, l_ping@jlu.edu.cn.

## Ethics Statement

The animal study was reviewed and approved by Ethics Committee of Jilin University.

## Author Contributions

JW and PL: conception and design. PL: administrative support. JW, YX, JH, JL, JX, BY, ZZ, BZ, HL, and PL: provision of study materials or patients and collection and assembly of data. JW: data analysis and interpretation. All authors: manuscript writing and final approval of manuscript.

## Conflict of Interest

The authors declare that the research was conducted in the absence of any commercial or financial relationships that could be construed as a potential conflict of interest.
